# Heliox reduces respiratory system resistance in respiratory syncytial virus induced respiratory failure

**DOI:** 10.1186/cc7880

**Published:** 2009-05-15

**Authors:** Martin CJ Kneyber, Marc van Heerde, Jos WR Twisk, Frans B Plötz, Dick G Markhors

**Affiliations:** 1 Department of Pediatric Intensive Care, VU university medical center, Amsterdam, The Netherlands; 2 Department of Pediatric Intensive Care, Beatrix Children's Hospital/University Medical Center Groningen, Groningen, The Netherlands; 3 Department of Biostatistics, VU university medical center, Amsterdam, The Netherlands

## Abstract

**Introduction:**

Respiratory syncytial virus (RSV) lower respiratory tract disease is characterised by narrowing of the airways resulting in increased airway resistance, air-trapping and respiratory acidosis. These problems might be overcome using helium-oxygen gas mixture. However, the effect of mechanical ventilation with heliox in these patients is unclear. The objective of this prospective cross-over study was to determine the effects of mechanical ventilation with heliox 60/40 versus conventional gas on respiratory system resistance, air-trapping and CO2 removal.

**Methods:**

Mechanically ventilated, sedated and paralyzed infants with proven RSV were enrolled within 24 hours after paediatric intensive care unit (PICU)admission. At T = 0, respiratory system mechanics including respiratory system compliance and resistance, and peak expiratory flow rate were measured with the AVEA ventilator. The measurements were repeated at each interval (after 30 minutes of ventilation with heliox, after 30 minutes of ventilation with nitrox and again after 30 minutes of ventilation with heliox). Indices of gas exchange (ventilation and oxygenation index) were calculated at each interval. Air-trapping (defined by relative change in end-expiratory lung volume) was determined by electrical impedance tomography (EIT) at each interval.

**Results:**

Thirteen infants were enrolled. In nine, EIT measurements were performed. Mechanical ventilation with heliox significantly decreased respiratory system resistance. This was not accompanied by an improved CO2 elimination, decreased peak expiratory flow rate or decreased end-expiratory lung volume. Importantly, oxygenation remained unaltered throughout the experimental protocol.

**Conclusions:**

Respiratory system resistance is significantly decreased by mechanical ventilation with heliox (ISCRTN98152468).

## Introduction

Respiratory syncytial virus (RSV) is the most important causative agent of lower respiratory tract disease (LRTD) in infancy [1]. Approximately 100,000 infants are annually admitted with RSV-induced bronchiolitis in the USA, and the number of hospitalizations is increasing [2]. Because of this, RSV-associated disease imposes a major burden on health care resources [3]. There is no effective therapy against RSV available, prevention can only be achieved through passive immunisation using monoclonal antibodies [4]. RSV LRTD is pathophysiologically characterized by sloughed necrotic epithelium, excessive mucus secretion, bronchial mucosal oedema and peribronchial inflammation that contributes to airway obstruction resulting in increased airway resistance with subsequent air-trapping and respiratory acidosis [5,6]. Although the majority of infections run a mild disease course, mechanical ventilation (MV) for up to 10 days is necessitated in approximately 2% to 16% of previously healthy hospitalised infants due to severe lower respiratory tract infection including bronchiolitis or pneumonia [1,7,8].

Helium is an inert gas with a density that is one-seventh that of air. In addition, carbon dioxide (CO_2_) diffuses more easily through helium than through air [9]. With helium, a more laminar flow is preserved in narrowed airways, resulting in lower resistance to gas flow allowing for increased bulk flow [10]. Based on these properties, MV with heliox could be considered in mechanically ventilated infants with RSV LRTD. Its use in these patients has been studied once but with inconclusive results [11].

We hypothesized that the use of heliox in mechanically ventilated infants with RSV LRTD would result in decreased respiratory system resistance (R_rs_). In addition, MV with heliox would result in less air-trapping defined by the relative change in end-expiratory lung volume (EELV), and improved CO_2 _clearance. The objective of our study was to test this hypothesis in a prospective, double cross-over intervention trial comparing heliox 60/40 with conventional gas (nitrox) using lung function testing and electrical impedance tomography (EIT) measurements.

## Materials and methods

### Patients

The study protocol (ISCRTN98152468) was approved by the hospital's Institutional Review Board and written informed consent was obtained from patients before enrollment.

Eligible for inclusion were infants younger than 12 months of age with a virologically confirmed clinical diagnosis of RSV LRTD (either a positive direct immunofluorescent assay or ELISA) who were admitted to the nine-bed paediatric intensive care unit (PICU) facility of the VU university medical center for MV during the RSV seasons (autumn and winter) between 2005 and 2007. Infants were excluded if no informed consent was obtained, fraction of inspired oxygen (FiO_2_) was more than 0.4, corticosteroids were used prior to admission, they were on high-frequency oscillatory ventilation or a haemodynamically significant congenital heart defect (i.e. significant left-to-right shunting with or without pulmonary hypertension) was present.

Patients were in supine position, intubated with an uncuffed endotracheal tube size 3.5 or 4.0 mm, and put on a time-cycled, pressure-limited ventilation mode (Pressure Control, AVEA ventilator, Cardinal Health, Yorba Linda, CA, USA). Aims of ventilation were transcutaneously measured oxygen saturation (SpO_2_) 88 to 92%, and partial pressure of arterial carbon dioxide (PaCO_2_) 45 to 65 mmHg (if pH >7.25). Inspiratory times were fixed at 0.5 seconds, positive end-expiratory pressure (PEEP) was set 1 to 2 cmH_2_O below total PEEP (i.e. extrinsic PEEP + intrinsic PEEP). The flow-time curve was observed thoroughly throughout the study period in each patient to examine if expiration was complete in order to prevent dynamic hyperinflation. Patients were sedated with midazolam and morphine, paralysis was achieved using intravenous rocuronium. Endotracheal suctioning was performed 30 minutes prior to the start of, but not during, the experimental protocol. Bronchodilators (either nebulized or intravenous) or ketamine were not used before or during the study period.

Arterial blood samples were drawn from an arterial line to determine PaCO_2 _and partial pressure of arterial oxygen (PaO_2_). End-tidal carbon dioxide (ET-CO_2_) concentration, and expiratory tidal volume (V_Te_) were measured at the airway opening. ET-CO_2 _was measured using a side-stream Microstream (Philips Medical Systems, Best, The Netherlands) and V_Te _was measured with a proximal flow sensor connected to the AVEA ventilator (Cardinal Health, Yorba Linda, CA, USA). The ventilator is designed to detect which gas is used and adjusts its pneumotachograph automatically in order to measure the correct V_Te_.

A chest radiograph was obtained and evaluated by one pediatric radiologist in each patient prior to the start of the experimental protocol to evaluate the presence of hyperinflation (defined by a depression of the diaphragm below the sixth anterior rib) or an infiltrate (described as opacities with irregular markings without loss of volume) [12].

### Experimental protocol

The experimental protocol started within 24 hours of PICU admission and lasted for 90 minutes. At four intervals (T = 0 (baseline), T = 30, T = 60, and T = 90 minutes) data were collected and respiratory variables measured. At T = 0 and T = 60, patients were ventilated with nitrox. At T = 30 and T = 90, patients were ventilated with heliox (helium 60%, oxygen 40%). Ventilator settings were kept constant throughout the experimental protocol.

Positive inspiratory pressure (PIP), intratracheal pressure (Ptrach), mean airway pressure (MAP), PEEP, SpO_2_, ET-CO_2_, respiratory rate and V_Te _were measured. Ptrach was measured with a pressure transducer placed at the distal end of the endotracheal tube. Blood samples were drawn for the determination of the PaO_2_, PaCO_2 _and pH. Static compliance (C_stat_), R_rs _and peak expiratory flow rate (PEFR) were measured using the AVEA ventilator (Cardinal Health, Yorba Linda, CA, USA) according to the manufacturer's manual. In summary, R_rs _was defined by the ratio of the airway pressure differential to the inspiratory flow 12 ms prior to the end of inspiration. Lung resistance (R_lung_) was defined by the ratio of the tracheal pressure differential to the inspiratory flow 12 ms prior to the end of inspiration.

### EIT measurements

At each interval, EIT measurements were made using the Göttingen Goe-MF II EIT system (Cardinal Health, Yorba Linda, CA, USA). Sixteen electrodes (Blue Sensor BR-50-K, Ambu, Denmark) were applied circumferentially around the infant's chest at the mammary line. A 30 second reference measurement at 13 Hz scan rate was recorded. All further measurements were referenced to this measurement. All other measurements were made at a scan rate of 44 Hz for 180 seconds. A 5 mA peak-to-peak, 50 kHz electrical current was injected at each adjacent electrode pair, and the resultant potential differences were measured at the remaining adjacent electrode pairs. Subsequently, all adjacent electrode pairs were used for current injection, thus completing one data cycle. The impedance map was built using the back-projection image reconstruction algorithm [13]. It calculates the relative impedance ΔZ, defined by (Z_inst _- Z_ref_)/Z_ref _(where Z_inst _is the instantaneous local impedance and Z_ref _the reference impedance, determined from each cycle of current injections and voltage measurements in each pixel).

### EIT data analysis

Both the respiratory and cardiac components of the EIT signal were identified in the frequency spectra generated from all EIT measurements (Fourier transformation). The EIT data was low-pass filtered with a cut-off frequency of 2 Hz to eliminate small impedance changes synchronous with the heart beat [14].

The calculations performed on the sums of values from all pixels of the 32 × 32 pixel matrix EIT image were described as 'global'. In addition, sums of values from the left and right lung regions were described separately, and the entire EIT image was divided into 64 regions-of-interest (32 left and 32 right lung) from anterior to posterior as previously described by Frerichs and colleagues [15]. Ventilation-induced tidal volume (ΔZ_VT_) was quantified by measuring the relative ΔZ from the highest point at end inspiration to the lowest point at end expiration, and an average ΔZ was calculated from multiple breaths. Changes in ΔZ_VT _were calibrated to volume using the known V_T_. The relative change in end-expiratory lung volume (relative ΔZ_EELV_) was determined by measuring the median impedance from the lowest point at expiration during the sampling time (Z_EELV_) [16]. The relative ΔZ_EELV _was normalized to volume (relative Δ_EELV _in ml) by multiplying the median impedance with the ratio V_T_/ΔZ_VT_.

### Calculation of respiratory indices and dead space

The oxygenation index (OI) was calculated as follows: (FiO_2 _× 100 × MAP in cmH_2_O)/PaO_2 _in mmHg. The ventilation index (VI) was calculated as follows: (PaCO_2 _in mmHg × respiratory rate × (PIP - PEEP in cmH_2_O))/1000. VI is used as determinant for CO_2 _elimination because the respiratory rate, PIP, and PEEP were kept constant throughout the study period [17]. Dead space (V_D_) was calculated according to the Bohr-Enghoff equation: V_D _= V_Te _× (1 - (P_ET-CO2_/PaCO_2_)) [18].

### Power analysis

As no data on relative Δ_EELV _in mechanically ventilated infants with RSV LRTD were available, we performed a power analysis after inclusion of all patients using the paired t-test.

### Statistical analysis

The data were analyzed with one-way repeated measures analysis-of-variance (ANOVA) with Tukey *post-hoc *testing between T = 0 versus T = 30, T = 30 versus T = 60, and T = 60 versus T = 90. *P *< 0.05 was accepted as being statistically significant. Data are expressed as mean ± standard deviation unless stated otherwise. Statistical analysis was performed using SPSS version 15.0 (Chicago, IL, USA).

## Results

**Figure 1:**
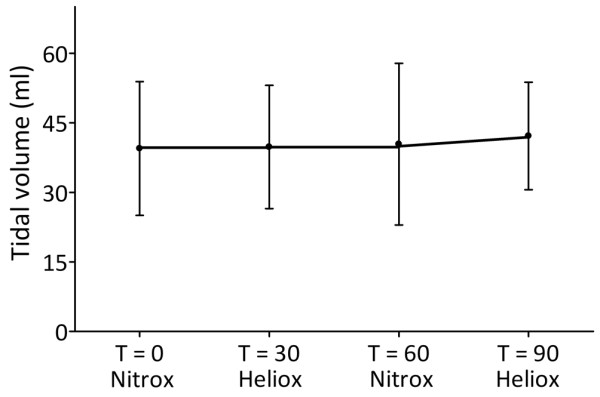
Course of tidal volume.

Thirteen patients were included in 11 EIT studies; good-quality EIT signals were obtained from nine patients. Descriptive data, ventilator settings and baseline data of respiratory system mechanics and gas exchange are summarized in Table 1. Although three patients were born prematurely (one at 32 weeks and two at 36 weeks' gestation), none of the patients had chronic lung disease. Hyperinflation was present in 10 patients, four of these patients also had infiltrates. Ten patients had hypercapnia (PaCO_2 _>45 mmHg) and seven infants had PaO_2_/FiO_2 _less than 200 at baseline (T = 0). Tidal volume remained constant throughout the experiment (Figure 1). Leakage around the uncuffed endotracheal tube was less than 5% in all patients.

**Table 1:** Descriptive data of the study population, ventilator settings and baseline characteristics of gas exchange and respiratory system mechanics

Pt	Age (weeks)	Gestational age (weeks)	Weight (kg)	Chest radiograph appearances	Baseline PaCO_2 _(mmHg)	Baseline PaO_2_/FiO_2 _^1^	PIP (cmH_2_O)	PEEP (cmH_2_O)	Baseline C_stat_ (mL/cmH_2_O/kg)	Baseline R_rs_ (cmH_2_O/L/sec)	Baseline PEFR (L/min)
*Patients without (full) EIT studies*											
1	11	Term	6.0	Hyperinflation + infiltrate	56	168	27	6	0.67	102.2	5.0
2	5	36	3.4	Infiltrate	46	346	26	10	0.29	92.9	13.0
3	3	Term	4.8	Hyperinflation	59	195	28	6	0.21	80.0	5.0
4	3	Term	3.7	Hyperinflation + infiltrate	59	188	26	6	0.27	58.1	4.0
											
*Patients with full EIT studies*											
5	4	Term	3.8	Hyperinflation + infiltrate	57	57	34	8	0.26	38.4	10.0
6	4	Term	4.3	Hyperinflation + infiltrate	75	265	32	5	0.47	28.6	5.0
7	23	Term	10.0	Hyperinflation	43	343	31	6	0.40	93.7	6.0
8	5	36	3.2	Infiltrate	35	140	32	7	0.31	53.6	5.0
9	15	Term	6	Hyperinflation	55	213	31	7	0.50	66.4	6.0
10	6	Term	5.4	Hyperinflation	49	418	29	7	0.19	94.8	7.0
11	11	32	3.5	Hyperinflation	68	295	33	5	0.29	51.0	4.0
12	6	Term	4.1	Hyperinflation	58	165	30	5	0.24	N/A	N/A
13	23	Term	7.5	Hyperinflation	44	170	24	11	0.40	69.8	6.0

^1^Fraction of inspired oxygen (FiO_2_) 0.4 in all patients.

C_stat _= static compliance; EIT = electrical impedance tomography; N/A = not available; PaCO_2 _= partial pressure of arterial carbon dioxide; PaO_2 _= partial pressure of arterial oxygen; PEEP = positive end-expiratory pressure; PEFR = peak expiratory flow rate; PIP = positive inspiratory pressure; Pt = patient; R_rs _= respiratory system resistance.

**Figure 2:**
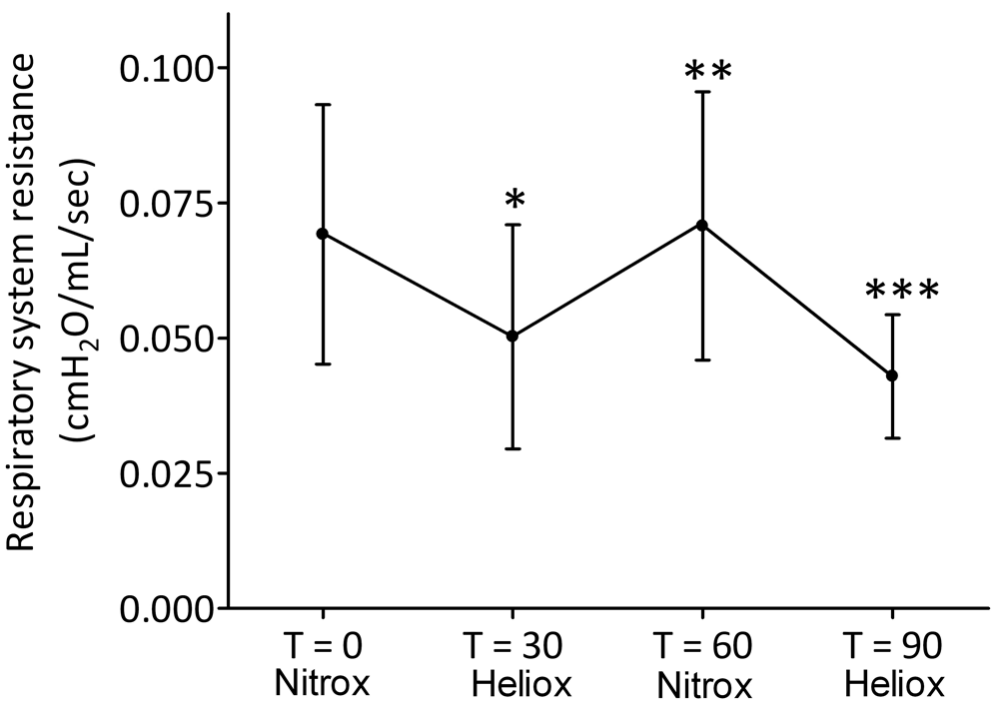
Effect of mechanical ventilation with heliox on respiratory system resistance. Data are expressed as mean ± standard deviation. * *P *< 0.05 T = 30 vs T = 0; ** *P *< 0.05 T = 60 vs T = 30; *** *P *< 0.05 T = 90 vs T = 60.

**Figure 3:**
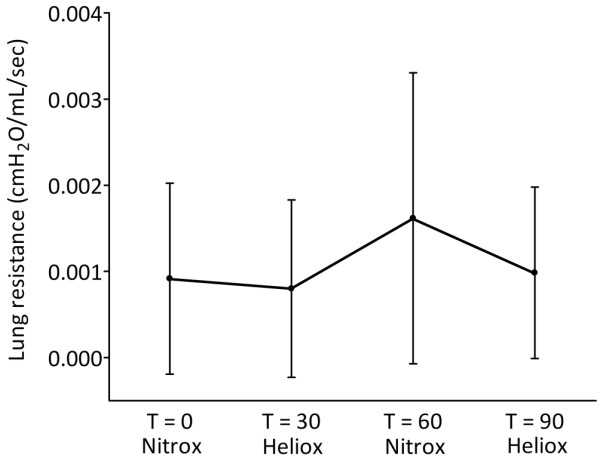
Effect of mechanical ventilation with heliox on lung resistance. Data are expressed as mean ± standard deviation.

Mechanical ventilation with heliox had an overall significant effect on R_rs _(*P *< 0.001; Figure 2). R_rs _decreased from 69.1 ± 6.9 cmH_2_O/L/sec at T = 0 to 50.2 ± 6.0 cmH_2_O/L/sec (*P *= 0.020) after 30 minutes of ventilation with heliox. After reintroduction of nitrox, R_rs _increased significantly to 70.7 ± 7.2 cmH_2_O/L/sec (*P *= 0.016) but decreased again to 42.9 ± 3.3 cmH_2_O/L/sec (*P *= 0.001) when heliox was reintroduced. R_lung _was not significantly influenced by MV with heliox (Figure 3).

**Figure 4:**
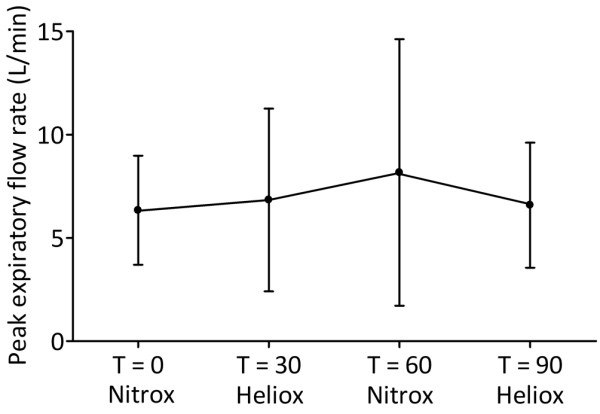
Effect of mechanical ventilation with heliox on peak expiratory flow rate. Data are expressed as mean ± standard deviation.

**Figure 5:**
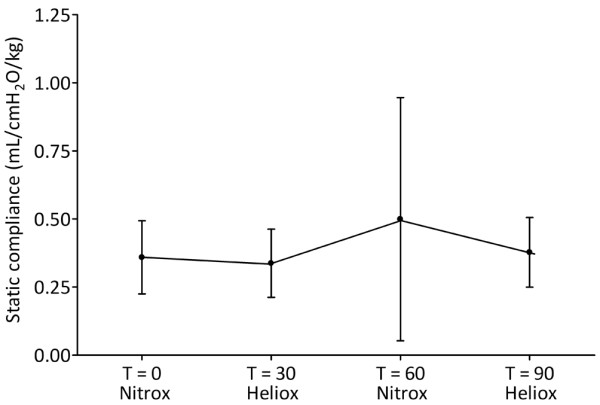
Effect of mechanical ventilation with heliox on static compliance. Data are expressed as mean ± standard deviation.

PEFR was not significantly improved by MV with heliox compared with nitrox (*P *= 0.520; Figure 4). C_stat _was 1.9 ± 0.4 L/cmH_2_O at T = 0 and not significantly different throughout the study (*P *= 0.214; Figure 5).

**Figure 6:**
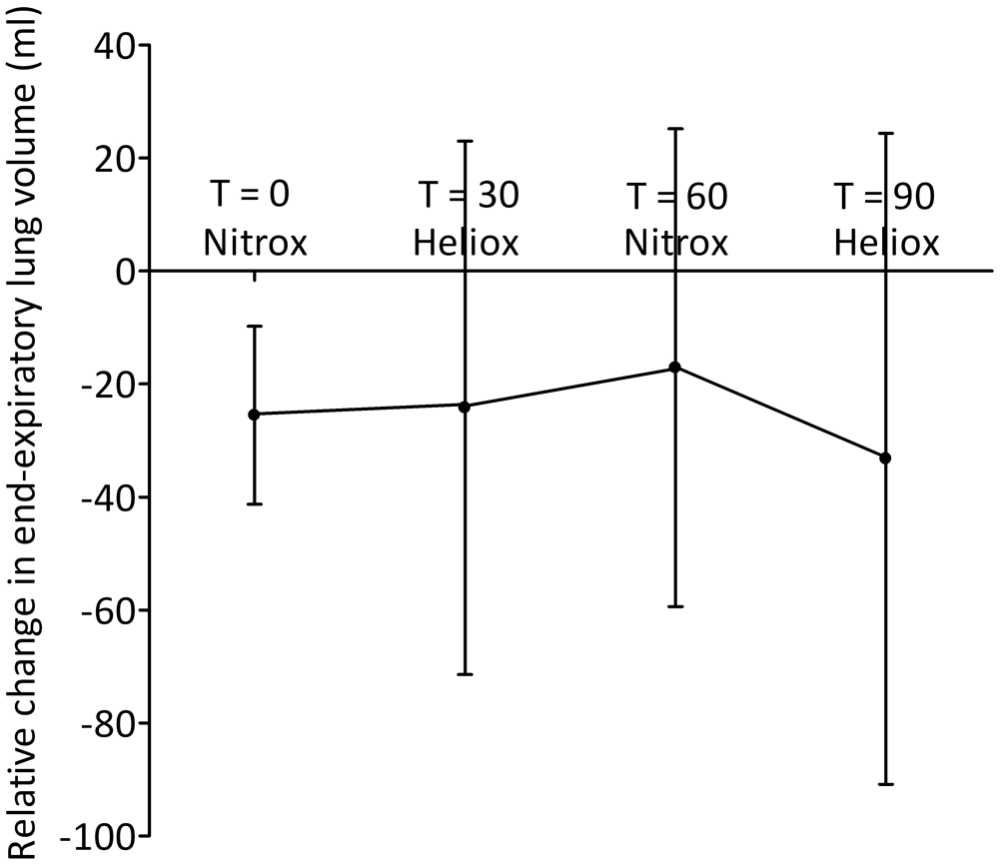
Effect of mechanical ventilation with heliox on relative change in end-expiratory lung volume. Data are expressed as mean ± standard deviation.

The mean relative Δ_EELV _± standard deviation at T = 0 was 76.6 ± 15.1 ml. With an estimated reduction of 25% with heliox, nine patients were needed to recruit in order to detect a statistically significant difference with α 0.05 and β 0.90. The degree of airtrapping as defined by the relative Δ_EELV _in ml was overall not significantly reduced by heliox (*P *= 0.493; Figure 6). This was due to differences in response to MV with heliox. Five patients showed a reduction in relative Δ_EELV _when heliox was introduced, and when conventional gas was reintroduced relative Δ_EELV _increased in only three patients (Table 2). There were also patients who had an increase of relative Δ_EELV _with heliox that was either reversed or increased when conventional gas was reintroduced.

**Table 2:** Response (%) of nine patients to mechanical ventilation with heliox or conventional gas as determined by EIT studies

Patient	Difference T = 30 to T = 0 (after heliox)	Difference T = 60 to T = 30 (after nitrox)	Difference T = 90 t0 T = 60 (after heliox)
5	0.5	12.9	-6.4
6	5.0	13.8	-7.7
7	5.1	4.5	-3.8
8	-39.0	12.4	-62.2
9	-19.6	20.3	2.3
10	-4.6	7.7	-21.9
11	0.0	-13.7	-7.5
12	0.3	4.9	0.0
13	42.1	-6.9	-3.8

Negative values indicate a decrease in relative change in end-expiratory lung volume (relative Δ_EELV_).

EIT = electrical impedance tomography.

To investigate if a time-dependent effect of heliox could be found, the change in relative Δ_EELV _was correlated with the change in R_rs _for T = 30 to T = 0 (R^2 ^0.068, *P *= NS), T = 60 to T = 30 (R^2 ^0.110, *P *= NS) and T = 90 to T = 60 (R^2 ^0.498, *P *= 0.01).

Fractional ventilation (i.e. the distribution between left and right lung), as well as the center of ventilation of the left and right lung, also remained constant throughout the study period (Table 3).

**Table 3:** Effect of mechanical ventilation with heliox on fractional ventilation, and center of ventilation as determined by electrical impedance tomography measurements

	Nitrox (T = 0)	Heliox (T = 30)	Nitrox (T = 60)	Heliox (T = 90)	*Overall* *P *value
*Fractional ventilation*					
Left lung (%)	50.8 ± 11.0	49.0 ± 10.9	50.1 ± 10.2	49.6 ± 11.2	0.65
Right lung (%)	49.2 ± 11.0	51.0 ± 10.9	49.9 ± 10.2	50.4 ± 11.2	0.65
*Center of ventilation*					
Left lung (%)	44.1 ± 8.0	42.8 ± 7.5	44.1 ± 7.7	43.6 ± 6.9	0.54
Right lung (%)	42.7 ± 6.4	41.6 ± 6.3	42.9 ± 7.0	43.0 ± 7.3	0.76

Data are expressed as percentages.

Table 4 summarizes the effect of mechanical ventilation with heliox on indices of gas exchange and V_D_/V_T_. Elimination of CO_2 _defined by the VI (*P *= 0.661), as well as a reduction in V_D_/V_T _(*P *= 0.929) was not positively influenced by MV with heliox. Importantly, oxygenation as defined by the OI (*P *= 0.477) and alveolo-arterial oxygen gradient (Aa-DO_2_) remained unaltered throughout the study period.

**Table 4:** Effect of mechanical ventilation with heliox on parameters for gas exchange and dead-space

	Nitrox (T = 0)	Heliox (T = 30)	Nitrox (T = 60)	Heliox (T = 90)	*Overall* *p *– value
OI	7.3 ± 6.0	6.8 ± 2.6	6.1 ± 2.1	6.6 ± 1.0	0.477
Aa-DO_2_	155 ± 135	131 ± 33	133 ± 68	134 ± 28	0.507
VI	44.8 ± 22.2	46.1 ± 22.6	48.3 ± 22.6	45.2 ± 18.9	0.601
V_D_/V_T_	0.20 ± 0.09	0.21 ± 0.11	0.20 ± 0.08	0.20 ± 0.11	0.929

Data are expressed as mean ± standard deviations.

Aa-DO2 = alveolo-arterial oxygen gradient; OI = oxygenation index; VI = ventilation index; V_D_/V_T _= dead-space/tidal volume ratio.

## Discussion

The major finding of our study is that MV of infants with RSV LRTD with heliox 60/40 resulted in a significant reduction of the respiratory system resistance.

Increased R_rs _resulting from airway narrowing due to sludging, excessive mucus secretion, edema, and possible bronchoconstriction has been described in mechanically ventilated infants with RSV LRTD [19-23]. Measures to alleviate increased R_rs _such as nebulisation of bronchodilators or nitric oxide have yielded inconclusive results [20,22,24,25]. However, these studies are methodologically different compared with ours. For instance, we excluded patients with chronic lung disease or congenital heart disease.

The decrease in R_rs _led not to an improved CO_2 _clearance as defined by the VI or a reduction in PEFR. Some explanations for this may be proposed. First, it is uncertain how much of the observed reduction in R_rs _could be partitioned to the ventilator circuit or the endotracheal tube because no endotracheal suctioning was performed during the study. Increased mucus production during RSV LRTD is common, and may further obstruct the airways [26]. As the AVEA ventilator is able to calculate the R_lung_, we also studied if MV with heliox resulted in a reduction in R_lung_, but were unable to demonstrate this. This could mean that MV with heliox does not affect the resistance of the small airways of the infants; it cannot be ruled out, however, that the resolution of the AVEA's signal of R_lung _(1 decimal) might not be sufficient enough to detect true differences in R_lung _in small children with little tidal volume. Second, the measured R_rs _in our patients is lower than previously reported in mechanically ventilated infants with RSV LRTD designated to have an obstructive disease phenotype [20,22,27]. This could indicate that our patients had mild-to-moderate airway obstruction, although hyperinflation suggesting airway obstruction on chest radiograph was present in all but one patient. Unfortunately, there is no gold standard for the radiological definition of hyperinflation especially in mechanically ventilated infants. Furthermore, the degree of air-trapping might vary between patients, indicating that severe RSV LRTD necessitating MV is a heterogeneous disease in which patients express to a varying degree both restrictive and obstructive disease characteristics explaining why some patients had a PaO_2_/FiO_2 _ratio of less than 200 or a C_stat _less than 0.3 ml/cmH_2_O/kg in our study. This assumption opposes the previously proposed dichotomization of RSV LRTD by Hammer and colleagues, who have observed that mechanically ventilated infants with RSV LRTD showed either a disease pattern compatible with acute respiratory distress syndrome (ARDS) or a disease pattern characterized by increased airway resistance [27]. Although our study was not designed to investigate differences in clinical phenotype, we would dare to challenge this dichotomy in clinical phenotype for several reasons. Hammer and colleagues included prematurely born infants with chronic lung disease and infants with congenital heart disease [27]. C_rs _is significantly lower in these patients compared with healthy infants [28-30]. In addition, the term 'bronchiolitis' to describe RSV LRTD is strictly speaking a histopathologic diagnosis and hampered by universal differences in its clinical interpretation [31]. Controversy exists about whether differences in parameters for gas exchange correlate with clinical phenotype [32,33].

The lack of improved CO_2 _clearance in our study is compatible with the observations by Gross and colleagues [11]. They were unable to demonstrate a beneficial effect on PaCO_2 _of various heliox mixtures (ranging from 50%/50% to 70%/30%) compared with T = 0 (PaCO_2 _45 ± 10 mmHg) in 10 mechanically ventilated infants with moderate severe RSV LRTD. It should be mentioned, however, that our study population was probably more ill than theirs based on a higher T = 0 PaCO_2 _and lower PaO_2_/FiO_2 _ratio. Previously, we did observe a beneficial effect of heliox in a small infant with obstructive airway disease [34]. This disparity in results cannot easily be explained except for the fact that this particular patient had severe respiratory acidosis.

EIT is a non-invasive bedside technique to assess global and regional lung volumes that has primarily been used in acute lung injury or ARDS [35]. Hinz and colleagues have shown that compared with the validated nitrogen-washout method it is an appropriate tool to study EELV in critically ill patients [16]. To our knowledge, the use of EIT in the determination of the dynamic process of air-trapping in patients with small airway disease has not been used before, although its use in this disease condition can be rationalised. In our study, MV with heliox did not result in a universal reduction of air-trapping as defined by the relative Δ_EELV_. However, there were some patients who seemed to benefit from MV with heliox as they did show a reduction in relative Δ_EELV _that was reversed by MV with conventional gas. Several explanations for the non-universal reduction in relative Δ_EELV _may be proposed. First, not all alveoli have the same degree of hyperinflation due to the difference in time constants throughout the lung, indicating that hyperinflation is a regional phenomenon rather than a global problem [36]. This would implicate that the technique of EIT may be insufficient to detect regional differences in viral-induced small airway disease due to heterogeneity of the disease, a problem that can be overcome by increasing the resolution of the EIT signal. In favor of EIT, however, is the study by Adler and colleagues showing that with EIT dynamic hyperinflation could be adequately monitored [37]. Second, during the study no endotracheal suctioning was performed. Increased mucus production could obstruct the airways, resulting in the collapse of alveoli that is reflected by a decrease in EELV. As tidal volume remained constant throughout the experiment, we think that not performing endotracheal suctioning did not influence our results (Figure 5). Third, if there is a difference in expression of clinical phenotype of RSV LRTD a universal response in relative Δ_EELV _would not be expected. Some patients responded with a decrease in relative Δ_EELV _whereas others did not in our study. Also, redistribution of ventilation within each lung or between the left and right lung was not significantly influenced by MV with heliox. This is in line with a heterogeneous clinical phenotype of RSV LRTD.

There are some limitations to our study that should be mentioned. First, the small sample size of our study. This sample size does not allow discrimination between responders and non-responders nor a categorization of clinical phenotype based on chest radiographs, but this should be the subject of further research. Second, patients were paralyzed throughout the study, thus prohibiting spontaneous breathing and mucus clearance by the patient itself. We choose to do so to eliminate any confounding effect of spontaneous breathing on the degree of dynamic hyperinflation in order to truly assess the effect of MV with heliox. However, our findings require re-evaluation in spontaneously breathing mechanically ventilated infants. Supportive therapy maintaining spontaneous breathing could very well be a key element while awaiting therapeutic modalities for mechanically ventilated infants with RSV LRTD [38]. Third, the measurements of our study were not blinded because connection of the heliox and the measurements were conducted by one investigator (MK). However, this might have introduced measurement bias. Fourth, ventilation with heliox may have influenced the tidal volume measurements of the AVEA ventilator. The AVEA is equipped with the Bicore CP100™ pulmonary mechanics monitor that has been validated previously [36,39]. Finally, the AVEA performs in a similar way with respect to tidal volume measurement when heliox is used [40,41].

## Conclusions

MV with heliox significantly reduced R_rs _in mechanically ventilated infants with RSV LRTD with a heterogenous effect on the degree of hyperinflation and CO_2 _elimination. These findings warrant further study in order to identify a subgroup of mechanically ventilated infants with RSV LRTD who might benefit from MV with heliox.

## Key messages

• MV with heliox decreases respiratory system resistance in RSV LRTD.

• MV with heliox does not reduce air-trapping in RSV LRTD.

• MV with heliox does not improve gas exchange in RSV LRTD.

• RSV LRTD may actually be a heterogeneous disease.

## Abbreviations

ANOVA: analysis of variance; ARDS: acute respiratory distress syndrome; CO_2_: carbon dioxide; Cstat: static compliance; EELV: end-expiratory lung volume; EIT: electrical impedance tomography; ELISA: enzyme-linked immunosorbent assay; ET-CO_2_: end-tidal carbon dioxide; FiO_2_: fraction of inspired oxygen; LRTD: lower respiratory tract disease; MAP: mean airway pressure; MV: mechanical ventilation; OI: oxygenation index; PaCO_2_: partial pressure of arterial carbon dioxide; PaO_2_: partial pressure of arterial oxygen; PEEP: positive end-expiratory pressure; PEFR: peak expiratory flow rate; PICU: paediatric intensive care unit; PIP: positive inspiratory pressure; Ptrach: intratracheal pressure; relative Δ_EELV_: relative change in end-expiratory lung volume; Rlung: lung resistance; Rrs: respiratory system resistance; RSV: respiratory syncytial virus; SPO_2_: oxygen saturation; V_D_: dead space; VI: ventilation index; Vte: expiratory tidal volume.

## Competing interests

The authors declare that they have no competing interests.

## Authors' contributions

MK designed and performed the study, performed the statistical analysis and wrote the manuscript. MvH assisted in performing the study. JT assisted in the statistical analysis and contributed to the writing of the manuscript. DM analyzed the EIT data and contributed to the writing of the manuscript.

## References

[B1] Hall CB (2001). Respiratory syncytial virus and parainfluenza virus. N Engl J Med.

[B2] Shay DK, Holman RC, Newman RD, Liu LL, Stout JW, Anderson LJ (1999). Bronchiolitis-associated hospitalizations among US children, 1980–1996. JAMA.

[B3] Leader S, Kohlhase K (2003). Recent trends in severe respiratory syncytial virus (RSV) among US infants, 1997 to 2000. J Pediatr.

[B4] Kneyber MC, Kimpen JL (2004). Advances in respiratory syncytial virus vaccine development. Curr Opin Investig Drugs.

[B5] McNamara PS, Smyth RL (2002). The pathogenesis of respiratory syncytial virus disease in childhood. Br Med Bull.

[B6] Aherne W, Bird T, Court SD, Gardner PS, McQuillin J (1970). Pathological changes in virus infections of the lower respiratory tract in children. J Clin Pathol.

[B7] Leclerc F, Scalfaro P, Noizet O, Thumerelle C, Dorkenoo A, Fourier F (2001). Mechanical ventilatory support in infants with respiratory syncytial virus infection. Pediatr Crit Care Med.

[B8] Guerguerian AM, Gauthier M, Lebel MH, Farrell CA, Lacroix J (1999). Ribavirin in ventilated respiratory syncytial virus bronchiolitis. A randomized, placebo-controlled trial. Am J Respir Crit Care Med.

[B9] Gupta VK, Cheifetz IM (2005). Heliox administration in the pediatric intensive care unit: an evidence based review. Pediatr Crit Care Med.

[B10] Papamoschou D (1995). Theoretical validation of the respiratory benefits of helium-oxygen mixtures. Respir Physiol.

[B11] Gross MF, Spear RM, Peterson BM (2000). Helium-oxygen mixture does not improve gas exchange in mechanically ventilated children with bronchiolitis. Crit Care.

[B12] Simpson W, Hacking PM, Court SD, Gardner PS (1974). The radiological findings in respiratory syncytial virus infection in children. Part I. Definitions and interobserver variation in the assessment of abnormalities on the chest X-ray. Pediatr Radiol.

[B13] Brown BH (2003). Electrical impedance tomography (EIT): a review. J Med Eng Technol.

[B14] Wolf GK, Grychtol B, Frerichs I, van Genderingen HR, Zurakowski D, Thompson JE, Arnold JH (2007). Regional lung volume changes in children with acute respiratory distress syndrome during a derecruitment maneuver. Crit Care Med.

[B15] Frerichs I, Dargaville PA, van Genderingen HR, Morel DR, Rimensberger PC (2006). Lung volume recruitment after surfactant administration modifies spatial distribution of ventilation. Am J Respir Crit Care Med.

[B16] Hinz J, Hahn G, Neumann P, Sydow M, Mohrenweiser P, Hellige G, Burchardi H (2003). End-expiratory lung impedance change enables bedside monitoring of end-expiratory lung volume change. Intensive Care Med.

[B17] Paret G, Ziv T, Barzilai A, Ben Abraham R, Vardi A, Manisterski Y, Barzilay Z (1998). Ventilation index and outcome in children with acute respiratory distress syndrome. Pediatr Pulmonol.

[B18] Enghoff H (1938). Volumen inefficax, Bemerkungen zur Frage des schadlichen Raumes. Upsala Lakareforen Forh.

[B19] Gauthier R, Beyaert C, Feillet F, Peslin R, Monin P, Marchal F (1998). Respiratory oscillation mechanics in infants with broncholitis during mechanical ventilation. Pediatr Pulmonol.

[B20] Hammer J, Numa A, Newth CJ (1995). Albuterol responsiveness in infants with respiratory failure caused by respiratory syncytial virus infection. J Pediatr.

[B21] Jefferson LS, Coss-Bu JA, Englund JA, Walding D, Stein F (1999). Respiratory system mechanics in patients receiving aerosolized ribavirin during mechanical ventilation for suspected respiratory syncytial viral infection. Pediatr Pulmonol.

[B22] Patel NR, Hammer J, Nichani S, Numa A, Newth CJ (1999). Effect of inhaled nitric oxide on respiratory mechanics in ventilated infants with RSV bronchiolitis. Intensive Care Med.

[B23] Smith PG, el-Khatib MF, Carlo WA (1993). PEEP does not improve pulmonary mechanics in infants with bronchiolitis. Am Rev Respir Dis.

[B24] Derish M, Hodge G, Dunn C, Ariagno R (1998). Aerosolized albuterol improves airway reactivity in infants with acute respiratory failure from respiratory syncytial virus. Pediatr Pulmonol.

[B25] Mallory GB, Motoyama EK, Koumbourlis AC, Mutich RL, Nakayama DK (1989). Bronchial reactivity in infants with acute respiratory failure with viral bronchiolitis. Pediatr Pulmonol.

[B26] Boogaard R, Hulsmann AR, van VL, Vaessen-Verberne AA, Yap YN, Sprij AJ, Brinkhorst G, Sibbles B, Hendriks T, Feith SW, Lincke CR, Brandsma AE, Brand PLP, Hop WCJ, de Hoog M, Merkus PJFM (2007). Recombinant human deoxyribonuclease in infants with respiratory syncytial virus bronchiolitis. Chest.

[B27] Hammer J, Numa A, Newth CJ (1997). Acute respiratory distress syndrome caused by respiratory syncytial virus. Pediatr Pulmonol.

[B28] Gappa M, Pillow JJ, Allen J, Mayer O, Stocks J (2006). Lung function tests in neonates and infants with chronic lung disease: lung and chest-wall mechanics. Pediatr Pulmonol.

[B29] Howlett G (1972). Lung mechanics in normal infants and infants with congenital heart disease. Arch Dis Child.

[B30] Yau KI, Fang LJ, Wu MH (1996). Lung mechanics in infants with left-to-right shunt congenital heart disease. Pediatr Pulmonol.

[B31] Isaacs D (1998). Is bronchiolitis an obsolete term?. Curr Opin Pediatr.

[B32] Tasker RC, Gordon I, Kiff K (2000). Time course of severe respiratory syncytial infection in mechanically ventilated infants. Acta Paediatr.

[B33] Kneyber MC, Bendelac M, Plötz FB (2007). Respiratory indices do not characterize clinical phenotype of respiratory syncytial virus lower respiratory tract disease. Am J Respir Crit Care Med.

[B34] Kneyber MC, van HM, Markhorst DG, Plotz FB (2006). Mechanical ventilation with heliox decreases respiratory system resistance and facilitates CO2 removal in obstructive airway disease. Intensive Care Med.

[B35] Wolf GK, Arnold JH (2005). Noninvasive assessment of lung volume: respiratory inductance plethysmography and electrical impedance tomography. Crit Care Med.

[B36] Blanch L, Bernabe F, Lucangelo U (2005). Measurement of air trapping, intrinsic positive end-expiratory pressure, and dynamic hyperinflation in mechanically ventilated patients. Respir Care.

[B37] Adler A, Shinozuka N, Berthiaume Y, Guardo R, Bates JH (1998). Electrical impedance tomography can monitor dynamic hyperinflation in dogs. J Appl Physiol.

[B38] Tasker RC (2008). CPAP and HFOV: different guises of the same underlying intensive care strategy for supporting RSV bronchiolitis. Intensive Care Med.

[B39] Petros AJ, Lamond CT, Bennett D (1993). The Bicore pulmonary monitor. A device to assess the work of breathing while weaning from mechanical ventilation. Anaesthesia.

[B40] Rogers M, Spearman CB (2003). Accuracy of volumes deliverd and monitored by the Viasys AVEA ventilator during heliox administration [abstract]. Resp Care.

[B41] Perino CD, Hess DR (2003). Heliox delivery using the AVEA ventilator [abstract]. Resp Care.

